# Methods for the Manipulation of Herpesvirus Genome and the Application to Marek’s Disease Virus Research

**DOI:** 10.3390/microorganisms9061260

**Published:** 2021-06-10

**Authors:** Yifei Liao, Kanika Bajwa, Sanjay M. Reddy, Blanca Lupiani

**Affiliations:** Department of Veterinary Pathobiology, College of Veterinary Medicine & Biomedical Sciences, Texas A & M University, College Station, TX 77843, USA; liao.yifei@tamu.edu (Y.L.); kbajwa@cvm.tamu.edu (K.B.)

**Keywords:** herpesvirus, genome manipulation, Marek’s disease virus, pathogenesis

## Abstract

Herpesviruses are a group of double-strand DNA viruses that infect a wide range of hosts, including humans and animals. In the past decades, numerous methods have been developed to manipulate herpesviruses genomes, from the introduction of random mutations to specific genome editing. The development of genome manipulation methods has largely advanced the study of viral genes function, contributing not only to the understanding of herpesvirus biology and pathogenesis, but also the generation of novel vaccines and therapies to control and treat diseases. In this review, we summarize the major methods of herpesvirus genome manipulation with emphasis in their application to Marek’s disease virus research.

## 1. Introduction

*Herpesviridae* is a large family of double-strand DNA viruses that infect a wide range of hosts, including humans and animals. Most herpesvirus infections are asymptomatic, except in very young and immunocompromised individuals. Members of the *Herpesviridae* family share a similar virion structure which consists of linear double-strand DNA, icosahedral capsid, tegument and envelope, and a similar life cycle which consists of both lytic and latent phases. Herpesviruses genome size ranges from ~124 to 259 kilobases (kb) and encode for ~70–200 proteins involved in various aspects of virus infection [1]. According to the current virus taxonomy released by the International Committee on Taxonomy of Viruses (ICTV), the *Herpesviridae* family consists of three subfamilies (*Alphaherpesvirinae*, *Betaherpesvirinae*, and *Gammaherpesvirinae*) and an unassigned species *Iguanid herpesvirus* 2. Eight herpesviruses were identified to infect humans, including herpes simplex virus 1 and 2 (HSV-1 and HSV-2, or human herpesvirus 1 and 2, HHV-1 and HHV-2) and varicella-zoster virus (VZV or HHV-3) which are members of the *Alphaherpesvirinae* subfamily; human cytomegalovirus (HCMV or HHV-5), human herpesvirus 6 (including two variants, HHV-6A and HHV-6B), and human herpesvirus 7 (HHV-7) are members of the *Betaherpesvirinae* subfamily; Epstein–Barr virus (EBV or HHV-4) and Kaposi’s sarcoma-associated herpesvirus (KSHV or HHV-8) are members of the *Gammaherpesvirinae* subfamily [2].

Marek’s disease (MD) is a neoplastic disease of chicken which was first reported by József Marek in 1907 [3]. The causative agent was later identified as Marek’s disease virus (MDV, also known as *Gallid alphaherpesvirus* 2, GaHV-2), which is classified into the *Mardivirus* genus and *Alphaherpesvirinae* subfamily. MDV isolates are further classified into mild (m), virulent (v), very virulent (vv) and very virulent plus (vv+) pathotypes based on their virulence. There are two additional closely related but distinct virus species to MDV, including MDV-2 or GaHV-3 and turkey herpesvirus (HVT, also known as *Meleagrid alphaherpesvirus* 1, MeHV-1); however, only MDV infection causes lymphoproliferative disease in chickens [4,5]. MD is the first naturally occurring tumor disease in any species that is prevented by the use of vaccines [6]. Since the early 1970s, HVT was widely used as vaccine to prevent MD because it is non-oncogenic and closely related to MDV [7,8,9]. Later, MDV-2 (SB-1 strain), a naturally avirulent virus, and a cell culture attenuated MDV (CVI988/Rispens strain) were developed, as vaccines, to control more virulent MDV strains [7,9,10,11,12]. However, since these vaccines only prevent disease but not infection and shedding of MDV, more virulent field strains have emerged [13]; thus, MD is still of great importance to poultry industry causing USD ~2 billion losses annually [7]. In recent years, taking advantage of genome manipulation methods, researchers have gained better knowledge of MDV gene functions and have developed several MD vaccine candidates, such as *meq* (**M**DV **E**coRI **Q**) and *vTR* (**v**iral **t**elomerase **R**NA) deletion mutant MDV [14,15,16,17].

In the past decades, scientists have developed advanced methods to manipulate the herpesvirus genome to precisely study the function of viral genes, and which has helped understand herpesvirus biology and pathogenesis, as well as the development of novel vaccines and antiviral drugs for prevention of herpesvirus infection. In this review, we will summarize the basic principles as well as advantages and disadvantages of five major methods, including temperature sensitive (ts) mutant, marker assisted site-directed mutagenesis, overlapping cosmid clones, infectious bacterial artificial chromosomes (BAC), and clustered regularly interspaced short palindromic repeats (CRISPR)/Cas9 system, and their applications and contributions to our understanding of herpesvirus biology, especially MDV.

## 2. Temperature Sensitive (ts) Mutant

**Figure 1:**
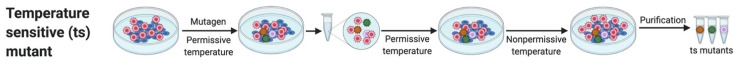
Schematic of generating temperature sensitive (ts) mutants.

A ts mutant is a type of conditional-lethal mutant that, due to the loss of normal protein functions induced by mutagens, can only grow at permissive temperatures but not at nonpermissive temperatures, while wild type virus can grow under both conditions. To induce mutations, virus infected cells were usually treated with chemical mutagens, such as 5-bromodeoxyuridine (BrdU) and nitrosoguanidine (NTG), or ultraviolet (UV) light. In the 1960s, generation of ts mutants or conditional-lethal mutants was widely used to study genetics and molecular biology of animal viruses [18,19,20]. In the 1970s, Schaffer et al. described the isolation of HSV-1 ts mutants and partially characterized the biochemical properties of those mutants [21,22,23]. Briefly, to generate ts mutants of a herpesvirus (Figure 1), the virus infected cells were first treated with chemical mutagens at permissive temperature to generate the mutant virus stocks, which were then co-seeded with cell monolayers at the permissive temperature for 2 days. After individual viral plaques appeared and outlined, infected cells were transferred to the nonpermissive temperature for another day. The plaques that did not increase in size after 24 h were considered to be potential ts mutants, were harvested and grown at the permissive temperature until cytopathic effects appeared (usually 2 days). Ts mutant candidates were titrated at both permissive and nonpermissive temperatures, and the mutants exhibiting reduced growth capacity at the nonpermissive temperature were plaque-purified and served as ts mutants. Using this method, Schaffer et al. isolated 22 ts mutants of HSV-1 and classified them into 15 complementation groups [23]. Later, a complementation study of HSV-1 and HSV-2 ts mutants carried out by 10 independent laboratories identified 23 and 20 essential genes for HSV-1 and HSV-2, respectively [24]. Similarly, cytolysis-resistant and drug-resistant mutants were also introduced to study the function of herpesvirus genes [25]. These technologies also contributed to the generation and characterization of mutants from other herpesviruses, such as pseudorabies virus (PRV) [26], VZV [27], and cytomegalovirus [28,29]. The use of ts mutants in MDV research has been limited because of the highly cell associated nature of the virus. However, a ts mutant MDV replicated poorly in vivo and failed to induce a protective immune response [30].

The advantages and disadvantages of this method are outlined in Table 1. While it is a useful technique to obtain a large number of mutant viruses at once, the mutation rate is relatively low and plaque isolation and purification are both labor-intensive and time-consuming procedures. In addition, due to the nature of how mutations are introduced, it is common for a ts mutant virus to carry multiple mutations, which makes it impossible to precisely attribute the observed phenotype to any specific gene.

## 3. Marker Assisted Site-Directed Mutagenesis

**Figure 2:**
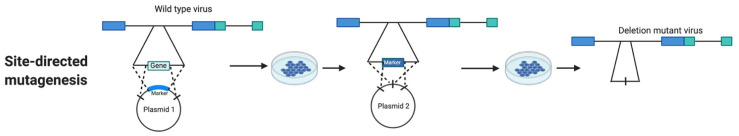
Schematic of marker assisted site-directed mutagenesis.

To overcome the shortcomings of ts mutants, taking advantage of DNA recombination, a more specific marker assisted site-directed mutagenesis method was developed for herpesvirus research. In the 1970s, scientists found that HSV-1 thymidine kinase (TK) could compensate for cellular TK activity in TK deficient mouse cells [31,32]. Further, the HSV-1 *Bam*HI digestion fragment containing the TK gene was cloned into *E. coli* plasmid pBR322 and the recombinant plasmid was transfected into mammalian cells to express TK [33]. Given the fact that TK is a dispensable gene for HSV-1 infectivity, and the presence of an efficient TK activity selection system, the TK gene was selected as the target for site-directed mutagenesis [34]. The above-mentioned recombinant plasmid (named pX1) together with HSV-1 DNA were used to generate a TK-deficient HSV-1 mutant in a two-step procedure [34]. In the first step, the pX1 plasmid was subjected to partial *Pst*I digestion, followed by ligation to generate pd2 plasmid where part of the TK gene was deleted; in the second step, the pd2 plasmid was cotransfected with nucleocapsids containing wild type HSV-1 genome into Vero cells to recover TK-deficient HSV-1 mutants [34]. Later, using TK as a selection marker, a generalized two-step site-directed mutagenesis technique was developed for HSV-1 genome manipulation [35]. Briefly, using a combination of DNA recombination strategy and selection media (hypoxanthine-aminopterin-thymidine (HAT) for selection of TK-proficient virus, and thymidine arabinoside (Ara T) for selection of TK-deficient virus), the TK gene was inserted as a selection marker into the gene of interest or in place of the gene of interest, in the first step, and then was deleted, in the second step (Figure 2). Using this technique, Post et al. deleted portions of HSV-1 infected cell polypeptide (ICP) 22 and found that ICP22 is dispensable for HSV-1 growth in cell culture [35].

Apart from TK, other markers, such as β-galactosidase (LacZ) and fluorescent proteins, were also used as selection or rescue markers in MDV studies (Table 2). Parcells et al. constructed a recombinant MDV mutant (named RB1BΔ4.5*lac*) by inserting the lacZ gene of *E. coli* into the unique short (U_S_) region of MDV resulting in the deletion of a 4.5 kb DNA fragment. The RB1BΔ4.5*lac* exhibited decreased in vitro growth, as well as impaired early cytolytic infection, horizontal transmission, tumor incidence and mortality in chickens, without affecting the latency or transformation of T lymphocytes [36] (Table 2). Using soluble-modified green fluorescent protein (smGFP) as selection marker, Parcells et al. generated a viral interleukin 8 (*vIL8*) deletion mutant MDV and showed that vIL8 is important for MDV lytic infection but dispensable for transformation [37].

The marker assisted site-directed mutagenesis opened the possibility for site specific manipulation of the herpesvirus genome and promoted the study of individual viral genes. However, due to the inefficient recombination process, it is common to result in a mixture of wild type and mutant viruses that needs further plaque purification steps (Table 1). Especially, for highly cell associated viruses like MDV, multiple rounds of plaque purification are needed which may result in the introduction of unexpected mutations. In addition, there is a possibility that the inserted foreign gene, such as lacZ or GFP, may affect the phenotype of the recombinant virus.

## 4. Overlapping Cosmid Clones

**Figure 3:**
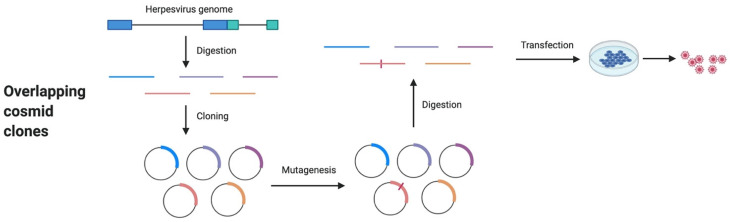
Generation of overlapping cosmid clones and their application to herpesvirus mutagenesis.

Cosmids (*cos* site + plasmid) are hybrid plasmids containing *cos* sequences of lambda phage and have been widely used for in vitro gene cloning since they were first reported in 1978 [66,67]. A cosmid vector usually contains an origin of replication (*ori*), a selection marker (e.g., antibiotic resistance gene), a *cos* site, and multiple cloning sites (MCS) for the insertion of a foreign DNA fragment of up to ~45 kb. Due to the large genome of herpesvirus, a series of overlapping cosmid clones (usually 4–5) are needed to cover the whole viral genome. In 1988, recombinant PRV mutants were successfully generated using five cosmid clones, containing overlapping DNA fragments of the PRV genome, and after cotransfection and subsequent recombination into permissive cells [68]. Briefly, the herpesvirus genome was digested with different restriction enzymes to generate overlapping viral DNA fragments which were then cloned into cosmid vectors. The gene of interest was then modified by deletion, mutation or insertion to generate the modified/recombinant viral cosmid. To reconstitute the mutant virus, all viral DNA fragments were released from the cosmids, through restriction enzyme digestion, and were cotransfected into permissive cells to produce the recombinant viruses (Figure 3). An advantage of this method is that it does not require plaque purification, because all reconstituted viruses are recombinant (contain the gene modification), allowing this technique to be widely used in herpesvirus research. Since originally introduced, this technique has been utilized to generate a large number of mutants for HSV-1, EBV, VZV, equine herpesvirus (EHV), MDV and HVT to study gene function [38,69,70,71,72,73,74].

The development of the overlapping cosmid technique significantly promoted research to study MDV gene function (Table 2). In 2002, a recombinant MDV was successfully generated using overlapping cosmid clones [38]. In this study, five overlapping MDV DNA fragments were generated from the vv Md5 strain of MDV after digesting with different restriction enzymes, which were cloned into cosmid vectors generating overlapping cosmid clones, named SN5, P89, SN16, A6, and B40. The MDV *pp38* gene, located in cosmid A6, was then deleted using a RecA-assisted restriction endonuclease (RARE) cleavage method. Finally, unmodified cosmids (SN5, P89, SN16, and B40) and mutated cosmid A6 (A6Δpp38) were digested and the five overlapping viral DNA fragments were purified and cotransfected into cells to produce the recombinant MDV with deletion of *pp38* gene (rMd5Δpp38). This recombinant virus showed that pp38 is important for MDV early cytolytic infection in lymphocytes, but dispensable for virus growth in cell culture, tumor formation in chickens and horizontal transmission of MDV [38,39]. Using the same technique, Cui et al. generated a recombinant Md5 virus in which *vIL8* was deleted and showed that vIL8 is important for MDV early cytolytic infection in lymphoid organs, but dispensable for establishment of latency and virus horizontal transmission [40,41]. In addition, they found this mildly virulent vIL8 deletion mutant protects against challenge with vv+ MDV in maternal antibody-positive chickens [41]. Most importantly, using overlapping Md5 cosmid clones, Lupiani et al. constructed an *meq* deletion virus in 2004 and found that Meq is essential for tumor formation but dispensable for virus replication in cell culture, lymphoid organs and epithelial cells of feather follicles, providing the first conclusive evidence that Meq is critical for MDV transformation of lymphocytes [17]. Using the same technique, Suchodolski et al. generated chimeric Md5 viruses by replacing the leucine zipper region of Meq with the leucine zipper region of yeast GCN4 and chicken c-Fos transcription factors [42,43]. This study showed that both homo- and heterodimerization properties of Meq are important for MDV induced transformation of lymphocytes [42,43]. Similarly, deletion of LORF11, an MDV unique gene, showed that it is important for MDV replication and pathogenesis in chickens [44].

The advantage of this method over those described earlier is the absence of required plaque purification step that could result in unintended mutations, which is especially important for the generation of recombinant highly cell associated viruses (Table 1). In addition, the overlapping cosmid approach does not rely on selection markers that are likely to interfere with virus replication. On the other hand, one limitation of this method is the handling of large DNA fragments as some cosmids are found to be unstable [75] and multiple recombination events are needed to reconstitute the full-length viral genome in cells, both of which may cause unwanted mutations and genome rearrangement in the resulting recombinant viruses (Table 1). In addition, it is difficult to construct revertant viruses, due to the nature of the method, a necessary step to rule out the possibility that the altered phenotype is due to other unwanted mutations. However, with the use of whole genome sequencing, the need for generation of revertant viruses could be avoided.

## 5. Infectious Bacterial Artificial Chromosome (BAC) Clones

In the early 1990s, a bacteriophage P1 and *E. coli* mini-F plasmid dependent cloning systems, named PAC (P1-derived artificial chromosome) and BAC (bacterial artificial chromosome), were developed as they are capable of maintaining large foreign DNA fragments; especially, a BAC can stably harbor up to 300 kb DNA sequences [76,77,78]. In addition, a BAC carrying full-length viral genomes can easily produce infectious viral particles by transfection into permissive cells. Thus, the BAC technique was rapidly adapted for virological studies. The circular genome of baculovirus was firstly cloned into a BAC vector and was proved to be an efficient method for expression of foreign proteins [79]. The first infectious BAC of herpesvirus was reported by Messerle et al., where the ~230 kb genome of mouse cytomegalovirus (MCMV) was cloned [80]. In addition, they successfully generated a recombinant virus carrying a mutated immediate-early 1 (IE1) gene and found that IE1 is important but not essential for MCMV growth in vitro [80]. To date, a large number of herpesviruses have been cloned into BAC vectors, facilitating every aspect of herpesvirus research [81,82].

**Figure 4:**
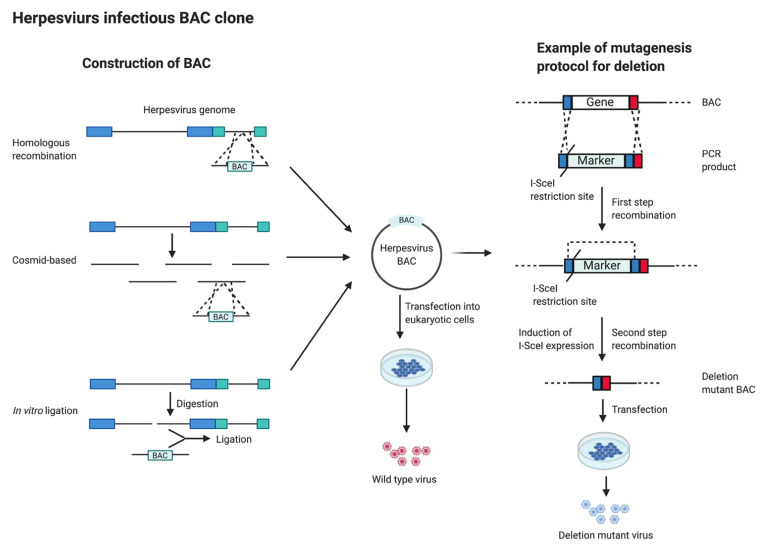
Generation of virus BAC clone and its application to herpesvirus mutagenesis.

To generate an infectious herpesvirus BAC, a cassette containing the mini-F factor and selection marker is inserted into the herpesvirus genome; thus, several methods (Figure 4), including homologous recombination, cosmid-based approaches and in vitro ligation, were developed [81,82]. The most widely used method is homologous recombination, which was used to generate the first MCMV BAC [80]. Briefly, the viral DNA and a transfer vector, which contains the mini-F factor and a selection marker flanked by sequences homologous to the insertion site in the viral genome, were cotransfected into mammalian cells for recombination. After selection, the circular viral DNA harboring BAC sequences were isolated and electroporated into *E. coli*. Alternatively, a cosmid-based approach was used to generate herpesvirus BACs as no selection steps are needed [83,84,85]. Similar to the insertion of a foreign gene using overlapping cosmid clones, the BAC sequence is inserted in one of the cosmid clones, followed by cotransfection to generate a BAC containing the entire viral genome. This method is convenient if the overlapping cosmid clones were already available for the virus. Direct in vitro ligation can also be a choice if a single restriction enzyme cutting site is available in the viral genome. This method was used successfully to construct a BAC clone for HHV-6A, where the viral DNA was digested with *Sfi*l (cut the viral genome in a single site) and ligated with BAC sequences [86]. However, this method has obvious drawbacks, such as the difficulty of finding a suitable restriction site and the low ligation efficacy of large DNA fragments.

After constructing the infectious BAC clone, manipulation of herpesvirus genome can be achieved by both random, such as transposon-mediated mutagenesis, and site-specific manners, such as RecA and Red/RecET-mediated mutagenesis [81,82,87]. In the random method, transposable elements (Tn) are randomly inserted into the viral BAC clone to disrupt viral genes, allowing for rapid generation of recombinant BAC libraries and global genome analysis. A Tn1721-based transposon system was developed to globally screen essential and nonessential genes of MCMV [88]. Similarly, a Tn5-based transposon mutagenesis was developed for rapid screening of nonessential genes for MDV replication in vitro [89]. For the purpose of introducing site-specific mutations, the RecA or Red-based homologous recombination systems have commonly been used. The RecA-mediated mutagenesis, also called shuttle mutagenesis, is achieved in a two-step procedure [81]. Briefly, a shuttle vector, harboring RecA, positive and negative selection markers, and the desired mutation flanked by sequences homologous (usually 500 bp to 3 kb) to the target site in the viral BAC, are transformed into a virus BAC-containing *E. coli*. Expression of RecA facilitates recombination between the shuttle vector and the virus BAC clone, followed by positive and negative selections to generate the desired markerless mutant virus BAC clone. However, this method has several limitations, such as the use of a negative selection marker that may cause undesired recombination events, and that the induction of RecA may cause instability of the BAC, often causing the loss of part of the viral genome.

Alternatively, the Red-mediated mutagenesis is a better option for site-specific manipulation of the herpesvirus genome, since it requires shorter homologous arms (30–50 bp) and it is rare to have undesired recombination events [81]. Briefly, a PCR product harboring a positive selection marker flanked by homologous sequences to the target site in viral BAC are electroporated into the virus BAC-containing *E. coli*, where recombination results in the integration of the positive selection marker into the virus BAC clone. Moreover, several other techniques, such as the Cre/loxP system, positive and negative selection markers, two-way selectable markers and self-excisable system, have been combined with the Red-based method to remove the unwanted BAC sequences [81,82,87,90]. In addition, using I-*Sce*I endonuclease, the en passant mutagenesis was developed to achieve a marker-less manipulation allowing all types of mutations (point mutation, deletion and insertion), using the GS1783 strain of *E. coli* which harbors chromosomal inducible Red and I-*Sce*I activities [91]. In this method, a PCR product harboring an 18 bp I-*Sce*I site and a positive selection marker, flanked by short homologous sequences, is used (Figure 4). After the above stated Red-mediated recombination, the induction of I-*Sce*I endonuclease cleave the I-*Sce*I site resulting in the second step recombination, which completely remove the positive selection marker and any other foreign sequences [91,92].

The introduction of BAC technology has largely facilitated MDV research (Table 2). The first MDV BAC clone was generated by Schumacher et al., using the homologous recombination-based method, where the BAC sequences were inserted to the U_S_2 non-essential locus of an avirulent, cell culture attenuated MDV strain 584Ap80C [45]. In addition, they successfully deleted 2 kb sequences from glycoprotein B (gB) and found that gB is essential for cell-to-cell spread of MDV in vitro [45]. Since then, infectious BAC clones have been constructed for numerous pathogenic MDV strains (RB-1B, GX0101, 814, Md5, 686), as well as MDV-2 and HVT [85,93,94,95,96,97,98]. Using infectious MDV BAC clones, several MDV genes and encoded RNAs have been shown to be important for MDV replication, tumorigenesis and horizontal transmission. It has been shown that MDV Meq interacts with C-terminal-binding protein (CtBP) through CtBP-interaction domain, PLDLS motif [46]. Deletion of the CtBP-interaction domain completely eliminated MDV induced tumors suggesting that interaction between Meq and CtBP is essential for MDV tumorigenesis [46]. MDV encoded vTR was also shown to be important for MDV lymphomagenesis as an MDV BAC clone with both copies of vTR deleted induced lower tumor incidence in chickens [16]. In addition, MDV encoded microRNA miR-M4, an ortholog of chicken miR-155, was also shown to be critical for MDV lymphomagenesis as deletion of miR-M4 from an MDV BAC clone resulted in 90% reduction of tumor incidence [47]. Other than genes involved in MDV tumorigenesis, several genes, including UL13 (encodes a viral protein kinase), UL44 (encodes glycoprotein C, gC), UL47 (encodes a tegument protein) and UL54 (encodes ICP27), have been identified to be essential for horizontal transmission of MDV [48,49,50,51,52,53]. MDV encodes a U_S_3 serine/threonine protein kinase, which is conserved among members of the *Alphaherpesvirinae* subfamily [99]. By deleting the entire U_S_3 gene or mutating its kinase active site, we and others showed that MDV U_S_3 is involved in de-envelopment of perinuclear virions, actin stress fiber breakdown, antiapoptosis, MDV replication and gene expression [54,55,56,57]. Even though most genes encoded by UL and US regions of MDV exhibit similar functions to their homologs in HSV-1, some MDV genes exhibit distinct functions. It has been reported that VP22 (encoded by UL49) is not required for in vitro growth of HSV-1, while it is essential for growth of MDV in cell culture [58]. In addition, this method has been used to construct fluorescent tagged viruses by fusing fluorescent protein to viral proteins, such as UL47 tegument protein, Meq and VP22, which are valuable models to study MDV biology and pathogenesis [59,60,61]. Apart from MDV biology and pathogenesis studies, the BAC technology has also aided in the development of novel MD vaccine candidates by deleting or mutating genes associated with tumorigenesis and pathogenesis [7]. Given the essential role of Meq in MDV tumorigenesis, a Meq deletion mutant virus is the most promising MD vaccine candidate to date; however, it retains the ability of parental MDV to cause lymphoid organ atrophy in chickens [15,17,100,101] and therefore cannot be approved as a vaccine. We recently generated an Meq and vIL8 double deletion mutant MDV using a 686BAC clone and found that it provided protection against challenge with vv+ MDV comparable to that of CVI988/Rispens vaccine [102]. Moreover, the Meq and vIL8 double deletion virus did not cause lymphoid organ atrophy in chickens, making it an excellent vaccine candidate to control vv+ MDV infection.

Overall, the BAC technique provided an easy and reliable method for herpesvirus genome manipulation, significantly promoting the study of herpesvirus genes function in the last two decades. It has many advantages over previous methods, since BAC can stably carry large foreign DNA sequences, it is easy to manipulate using bacteria genetics, it can achieve both random and site-specific mutagenesis and it is easy to generate revertant BAC clones (Table 1). However, some viral DNA are unstable in BACs and BAC DNA may shear during the manipulation process, screening of recombinant BAC constructs can be labor-intensive and some unwanted recombination events or random mutations may occur (Table 1). It has been reported that a recombinant RB-1B, reconstituted from a BAC clone, was unable to transmit horizontally [103,104]. By comparing genome sequences of this recombinant RB-1B with published MDV genome sequences, frameshift mutations were identified in several MDV genes [103,104]. Subsequently, restoration with wild-type genes, via en passant mutagenesis, demonstrated that UL13 and gC are critical for efficient horizontal transmission of MDV [48,50].

## 6. Clustered Regularly Interspaced Short Palindromic Repeats (CRISPR)/Cas9 System

**Figure 5:**
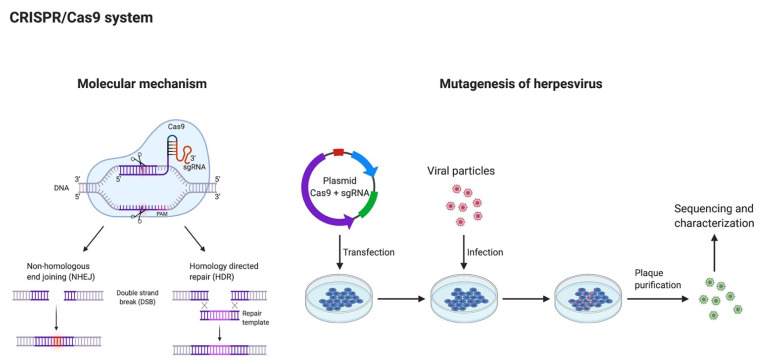
CRISPR/Cas9 system and its application to herpesvirus mutagenesis.

In the past decade, the CRISPR/Cas system has been extensively studied and proved to be a reliable tool for genome editing and a promising therapy strategy for various diseases. The CRISPR/Cas, an RNA-guided nuclease system, is a type of adaptive immunity of archaea and bacteria [105,106]. Currently, six types of CRISPR/Cas have been identified and are grouped into two classes: class 1 which consists of type I, III, and IV CRISPR systems that are characterized by multi-subunits of effector complexes; class 2 consists of type II, V, and VI CRISPR systems that are characterized by a single effector nuclease [107]. The most widely used type II CRISPR/Cas9 system includes three key components, CRISPR RNA (crRNA), trans-activating crRNA (tracrRNA) and Cas9 endonuclease, where the crRNA and tracrRNA are usually fused to a single-guide RNA (sgRNA) in practical use [108,109,110,111]. The sgRNA binds and recruits Cas9 to the target site that locates upstream of a three-nucleotides long protospacer adjacent motif (PAM) to create a DNA double stranded break (DSB), which could be repaired by non-homologous end joining (NHEJ) or homology directed repair (HDR) (Figure 5). Since the CRISPR/Cas9 system has been proved to be an efficient RNA-guided genome editing method in mammalian cells, it has also been adopted for large DNA virus research [108,111,112]. The CRISPR/Cas9 system was reported to successfully edit the genome of adenovirus, HSV-1 and EBV by different research groups in 2014 [113,114,115], and since then, numerous other herpesviruses have been shown to be efficiently edited using the CRISPR/Cas9 system [112,116]. Unlike the complexity of previous methods, scientists just need to design a 20 bp long RNA fragment complementary to the target site, which will be cloned into an all-in-one plasmid containing Cas9, sgRNA scaffold and selection marker. To edit the herpesvirus genome, permissive cells are transfected with the guide RNA (gRNA) and Cas9 expression plasmid, followed by infection with the virus. If precise editing is desired, a repair template will need to be cotransfected with the gRNA and Cas9 expression plasmid. After selection and purification, the modified herpesvirus genome is isolated and mutation confirmed by sequencing (Figure 5). Apart from its use in genome manipulation, the powerful editing ability of CRISPR/Cas9 system has been applied to study host–virus interactions, abrogate virus replication and develop novel therapies for herpesvirus infection, which are not the focus of this review and have been extensively reviewed elsewhere [112,116,117,118,119].

The CRISPR/Cas9 system has also been shown to be applicable to the manipulation of the MDV genome and to study MDV gene function (Table 2). In 2018, Zhang et al. successfully generated *pp38* or *meq* deletion CVI988 mutants using the CRISPR/Cas9 system [62]. MDV is a highly cell associated virus, thus the first-round of plaques normally contain a mixture of edited and unedited viruses, which can be further separated by another round of plaque purification. In this study, Zhang et al. showed that transfection of a pair of gRNAs that target the N- and C-terminal ends of *pp38* resulted in 12.5% genome editing efficiency; however, for *meq*, the editing efficiency varied from 25% to 75% as different gRNAs targeting the C-terminus were used [62]. Later, CRISPR/Cas9 system was further applied to edit *pp38* and miRNAs in MDV transformed lymphoblastoid cell line [63,64,65], providing an applicable and efficient method to study the MDV tumorigenesis and MDV–host interactions in vitro. Other than gene function studies, CRISPR/Cas9 technology mediated genome editing has also been used for the development of HVT vector vaccines. The first attempt was made by Tang et al., who used the CRISPR/Cas9 system to construct a recombinant HVT vector vaccine (HVT-VP2) by inserting the VP2 gene of infectious bursal disease virus (IBDV) into the UL45/46 locus of the HVT genome [120]. In a subsequent study, they inserted two additional viral antigen expression cassettes, which included genes of infectious laryngotracheitis virus (ILTV) and avian influenza virus (AIV), into the HVT-VP2 genome [121]. Theirs and other studies demonstrated that CRISPR/Cas9 is a simple and efficient method for the generation of vector vaccines carrying more than one foreign viral gene [122,123]. In addition, the nature of the CRISPR/Cas9 system promises the possibility of editing several target sites simultaneously, which will facilitate the development of recombinant vaccines that confer protection against multiple diseases. The CRISPR/Cas9 system has also been shown to be an efficient method to abrogate MDV or other virus infections. Hagag et al. showed that combination of two or more gRNAs that target MDV essential genes could completely abrogate MDV replication in cell culture [124]. In addition, MDV has been developed as a CRISPR/Cas9 delivery system to disrupt avian leukosis virus (ALV) infection in cell culture and chickens [125]. Recently, transgenic chickens constitutively expressing Cas9 and gRNA targeting ICP4, were generated, resulting in significant reduction of MDV replication, suggesting a novel antiviral method to restrict MDV replication [126].

Overall, the CRISPR/Cas system has taken the genome manipulation and other studies of herpesvirus biology into a new era. It has numerous advantages over previous herpesvirus genome manipulation methods, such as its ease of use, the absence of need of intermediate BAC constructs thus absence of BAC sequences in the virus genome, it allows the simultaneous modification at several sites/genes, it has various and powerful tool kits that fulfill different purposes, provides promising therapeutic potential for treating herpesvirus infection, and more (Table 1). However, the CRISPR/Cas9 system also has some limitations. The need of PAM sequences may limit the target sites, the off-target potential may cause unwanted mutations and may need multiple rounds of plaque purification to isolate the edited virus (Table 1).

## 7. Conclusions

From ts mutant to CRISPR/Cas9 system, the methods for herpesvirus genome manipulation have evolved from unspecific and laborious to precise and efficient editing, greatly facilitating our understanding of the biology and pathogenesis of herpesvirus. The introduction of overlapping cosmid clones and BAC technology accelerated the precise analysis of MDV gene function, promoted the study of molecular mechanisms of MDV oncogenesis and the development of recombinant MD vaccines. With the well-established genome manipulation system and small animal model (chicken), MDV has served as a very good model to study viral oncogenesis in its natural host. In recent years, the development of the CRISPR/Cas9 system has propelled the next stages of MDV research, though its use in MDV genome manipulation is still in early stages. Moreover, the CRISPR/Cas9 system has broader applications in the study of MDV–host interactions and novel vaccine development. Next generation sequencing has also played a central role in MDV genome manipulation and other studies. In combination with BAC and CRISPR/Cas9 systems, next generation sequencing will ensure accurate genome editing and comprehensive analysis of global changes in viral genes and host pathways, which will promote our understanding of the molecular mechanisms of MDV pathogenesis and the development of novel MDV vaccines.

## Figures and Tables

**Table 1:** Pros and cons of herpesvirus genome manipulation methods.

	Temperature Sensitive (ts) Mutant	Marker Assisted Site-Directed Mutagenesis	Overlapping Cosmid Clones	BAC Clone (RecA or Red-Based Homologous Recombination)	CRISPR/CAS9 System
Pros	1. Allows to generate large number of mutants at once	1. Allows site specific manipulation	1. Allows site specific manipulation 2. No selection and purification steps are needed	1. Capable of harboring large DNA fragments 2. Easy to manipulate using bacteria genetics 3. Can achieve both random and site-specific mutations 4. Easy to generate revertant BAC	1. Easy to handle and efficient 2. Will not retain BAC sequences in the virus genome 3. Allows the simultaneous manipulation at different sites 4. Provides therapeutic potential for treatment
Cons	1. Mutation frequency is low 2. Procedure is laborious 3. Difficult to precisely map the mutation site	1. Low recombination efficacy 2. Need plaque for the purification 3. The inserted foreign gene may affect phenotype of the recombinant virus	1. Difficult to handle large DNA fragments 2. Unwanted mutations may be introduced due to multiple recombination events 3. Difficult to construct revertant virus	1. The large BAC DNA may shear during the manipulation process (mostly with RecA) 2. Unwanted recombination events or random mutations may occur (mostly with RecA)	1. The need of PAM sequences may limit the target sites 2. The possibility of off-target may cause unwanted mutations 3. Needs multiple rounds of plaque purification

**Table 2:** Selected studies of MDV using different genome manipulation methods.

Method	MDV Strain	Manipulation	Main Findings	References
Marker assisted site-directed mutagenesis	RB-1B (vv)	Deletion of 4.5 kb sequences in U_S_ region of MDV genome	These genes are involved in virus replication, horizontal transmission, tumor formation, but not transformation	[36]
	RB-1B (vv)	Deletion of vIL8	vIL8 is important for MDV lytic infection but dispensable for transformation	[37]
Overlapping cosmid clones	Md5 (vv)	Deletion of pp38	pp38 is important MDV early cytolytic infection in lymphocytes but dispensable for virus growth in vitro, tumor formation in chickens and virus horizontal transmission	[38,39]
	Md5 (vv)	Deletion of vIL8	vIL8 is important for MDV early cytolytic infection in lymphoid organs, but dispensable for establishment of latency and virus horizontal transmission	[40,41]
	Md5 (vv)	Deletion of Meq	Meq is essential for tumor formation but dispensable for virus replication	[17]
	Md5 (vv)	Chimeric Meq mutants	Both homo- and heterodimerization of Meq are important for transformation of lymphocytes	[42,43]
	Md5 (vv)	Deletion of LORF11	LORF11 is important for MDV replication and pathogenesis in chickens	[44]
BAC clone	584Ap80C (vv+, attenuated)	Deletion of 2 kb sequences in gB	gB is essential for cell-to-cell spread of MDV in vitro	[45]
	RB-1B (vv)	Deletion of CtBP interaction domain in Meq	Meq-CtBP interaction is essential for MDV tumorigenesis	[46]
	RB-1B (vv)	Deletion of vTR	vTR is important for MDV induced T cell lymphoma	[16]
	RB-1B (vv)	Deletion of cluster 1 miRNAs and miR-M4	Cluster 1 miRNAs, especially miR-M4, are important for MDV induced T cell lymphomas	[47]
	RB-1B (vv)	Deletion or mutation	UL13, UL44, UL47 and UL54 are essential for horizontal transmission of MDV	[48,49,50,51,52,53]
	584Ap80C (vv+, attenuated), 686 (vv+)	Deletion of U_S_3 and mutation of U_S_3 kinase active site	U_S_3 is involved in de-envelopment of perinuclear virion, actin stress fiber breakdown, antiapoptosis, MDV replication and gene expression	[54,55,56,57]
	584Ap80C (vv+, attenuated)	Deletion of UL46 to UL49	UL46, UL47 and UL48 genes are nonessential, but UL49 is essential, for growth of MDV	[58]
	RB-1B (vv), Md5 (vv)	Fusing of fluorescent protein to UL47, Meq and VP22	Constructed fluorescent tagged viruses, which are valuable models to study MDV biology and pathogenesis	[59,60,61]
CRISPR/Cas9 system	CVI988 (vaccine strain)	Deletion of Meq and pp38	CRISPR/Cas9 system is applicable for MDV genome manipulation and gene function study	[62]
	MDV transformed lymphoblastoid cell line	Deletion of pp38 and miRNAs	CRISPR/Cas9 system is applicable for MDV genome manipulation in MDV lymphoblastoid cell line	[63,64,65]

## Data Availability

Not applicable.
